# Aggressive Squamoid Eccrine Ductal Carcinoma of the Face: A Rare and Challenging Diagnosis—Case Report and Literature Review

**DOI:** 10.3390/medicina61040612

**Published:** 2025-03-27

**Authors:** Bruno Špiljak, Damir Sauerborn, Matej Tomas, Brankica Gregorić Butina, Ivana Mahovne, Suzana Erić, Bruno Vidaković, Stjepanka Lešić

**Affiliations:** 1 Department of Oral Medicine, University of Zagreb School of Dental Medicine, 10000 Zagreb, Croatia; bspiljak@sfzg.unizg.hr; 2 Department of Otorhinolaryngology and Head and Neck Surgery, General Hospital “Dr. Josip Benčević”, 35000 Slavonski Brod, Croatia; dsauerborn@fdmz.hr (D.S.); brane.greg@gmail.com (B.G.B.); 3 Department of Dental Medicine, Faculty of Dental Medicine and Health Osijek, Josip Juraj Strossmayer University of Osijek, 31000 Osijek, Croatia; mtomas@fdmz.hr; 4 Department of Pathology and Cytology, General Hospital “Dr. Josip Benčević”, 35000 Slavonski Brod, Croatia; i.mahovne@yahoo.com; 5 Department of Oncology, Clinical Hospital Centre, 31000 Osijek, Croatia; seric@fdmz.hr

**Keywords:** squamoid eccrine ductal carcinoma, cutaneous adnexal tumor, aggressive skin neoplasm, perineural invasion, Mohs micrographic surgery, adjuvant radiotherapy

## Abstract

*Background*: Squamoid eccrine ductal carcinoma (SEDC) is an exceedingly rare and aggressive cutaneous adnexal malignancy, with fewer than 100 reported cases. Its histopathologic overlap with squamous cell carcinoma (SCC) frequently leads to misdiagnosis, delaying appropriate management. Unlike SCC, SEDC exhibits biphasic differentiation, deep infiltration, and a high rate of perineural invasion, contributing to significant morbidity and poor long-term outcomes. Given the absence of standardized treatment protocols, managing SEDC remains a challenge. *Case Presentation*: We report an unusual case of an 80-year-old female presenting with progressive numbness, nasal deviation, and a subcutaneous indurated lesion in the left nasofacial region. The early neurological symptoms were an atypical feature, suggesting perineural invasion (PNI) before visible tumor progression. Initial histopathologic evaluation was inconclusive, raising suspicion of SCC, necessitating immunohistochemical analysis, which confirmed ductal differentiation, leading to the final diagnosis of SEDC. The patient underwent radical resection with intraoperative margin assessment (Mohs micrographic surgery; MMS) followed by adjuvant radiotherapy (62 Gy/31 fractions) due to high-risk features, including perineural and perivascular invasion. Despite initial disease control, a local recurrence involving the left orbit and nasal bone occurred 20 months postoperatively, demonstrating the aggressive nature of SEDC despite clear surgical margins and adjuvant therapy. Due to disease progression and refusal of further surgery, only palliative care was provided. During follow-up, the patient contracted COVID-19, further complicating her clinical status and contributing to her demise. While COVID-19 was not directly linked to SEDC progression, its impact on patient management was significant. *Conclusions*: This case underscores the diagnostic and therapeutic challenges of SEDC, emphasizing the need for early suspicion, extensive histopathologic assessment, and aggressive multimodal treatment. The importance of multidisciplinary management—particularly in elderly and immunocompromised patients—and long-term surveillance due to high recurrence risk and PNI is crucial.

## 1. Introduction

Squamoid eccrine ductal carcinoma (SEDC) is an exceptionally rare malignant adnexal tumor with eccrine and apocrine differentiation, first described by Wick and Swanos [1] in 1991 as a subtype of eccrine carcinoma with squamous differentiation. In 1997, Wong et al. [2] introduced the term SEDC after reporting three cases, highlighting its biphasic histopathological nature, which frequently leads to misdiagnosis as squamous cell carcinoma (SCC), particularly in superficial biopsies. SEDC accounts for less than 0.01% of all malignant cutaneous neoplasms, making it one of the rarest adnexal carcinomas [2,3,4,5,6]. Clinically, SEDC often presents as ulcerations, nodules, or plaques, predominantly in sun-exposed areas, particularly the head and neck region [4,7]. It primarily affects elderly individuals, with a male predilection and peak incidence in the seventh to eighth decade of life [4,8]. Immunosuppression has been suggested as a potential risk factor, although further studies are required to confirm this association [9]. Histopathologically, SEDC exhibits an infiltrative growth pattern with poorly demarcated margins, extending deeply into the dermis and subcutaneous tissue. The tumor’s superficial regions typically display squamous differentiation, while deeper portions exhibit ductal structures characteristic of eccrine differentiation [2,4]. Actinic keratosis has been associated with SEDC in 86% of reported cases, suggesting a potential link between chronic sun exposure and tumorigenesis [4]. The rarity of SEDC and its histological resemblance to SCC frequently lead to diagnostic delays, with dermoscopic findings inconsistently reported and of limited diagnostic value [10,11]. Due to its biphasic nature, approximately 50% of cases are initially misdiagnosed as SCC, as superficial biopsies capture only the squamous component [4,12]. A definitive diagnosis is often made only after complete excision when the deeper eccrine ductal differentiation becomes evident [13]. Immunohistochemical analysis aids in distinguishing SEDC from SCC and other adnexal tumors, with SEDC typically expressing epithelial membrane antigen (EMA) and carcinoembryonic antigen (CEA) in ductal regions, a staining pattern absent in SCC [4,14]. Generally, malignant sweat gland tumors are broadly categorized into high- and low-grade malignancies, with high-grade tumors exhibiting significant metastatic potential and disease-related mortality, whereas low-grade tumors primarily demonstrate locally aggressive growth with lower metastatic rates [15]. SEDC has traditionally been classified as a low-grade malignancy, along with microcystic adnexal carcinoma, whereas aggressive digital papillary adenocarcinoma and spiradenocarcinoma are considered high-grade malignancies [15]. Although historically classified as a low-grade malignancy, SEDC demonstrates aggressive local behavior, with recurrence rates reaching 25% and metastatic spread occurring in 13% of cases [4]. The tumor’s capacity for perineural and perivascular invasion further contributes to its high recurrence rate, independent of surgical margin status [4]. Given these aggressive features, the traditional classification of SEDC as a low-grade tumor has been challenged. Given its aggressive nature and high recurrence risk, wide local excision remains the preferred treatment approach. Mohs micrographic surgery (MMS) has demonstrated the best outcomes, particularly for facial lesions, as it allows precise margin control while preserving healthy tissue [8,14]. However, due to the limited number of reported cases and the absence of standardized treatment guidelines, long-term disease monitoring is essential to optimize patient outcomes. This case report and literature review aim to enhance awareness of this rare malignancy, highlight its diagnostic challenges, and contribute to the evolving understanding of SEDC. Moreover, this case highlights an unusual presentation of SEDC, with early neurological symptoms such as facial numbness, preceding any overt cutaneous manifestations. This suggests that perineural invasion (PNI) might play a critical role in early disease progression, serving as an important yet under-recognized early diagnostic clue. Additionally, despite aggressive surgical resection with intraoperative margin assessment (MMS) and adjuvant radiotherapy, the patient experienced a rapid local recurrence with orbital invasion. This case underscores the need for heightened clinical suspicion, particularly in cases with atypical neurological involvement, and reinforces the importance of long-term surveillance even after aggressive treatment.

## 2. Case Report

### 2.1. Clinical Examination

An 80-year-old female patient was referred to our clinic with a symptom of numbness in the area of the left eyebrow and left half of the nose, with occasional burning sensations in the same area. In the last couple of months, she noticed a “turning” of the nose to the left side and the appearance of a subcutaneous formation on the lateral aspect of the left side of the nose. On physical examination, the patient had a deviation of the nose to the left side and indurated skin at the lateral aspect of the left side of the nose below the medial canthus, with a subcutaneous hardening measuring 1 × 1.5 cm. Intranasal examination showed a deviation of the nasal septum to the right side, partially obstructing the left nasal cavity.

### 2.2. Imaging and Preoperative Diagnostics

**Figure 1:**
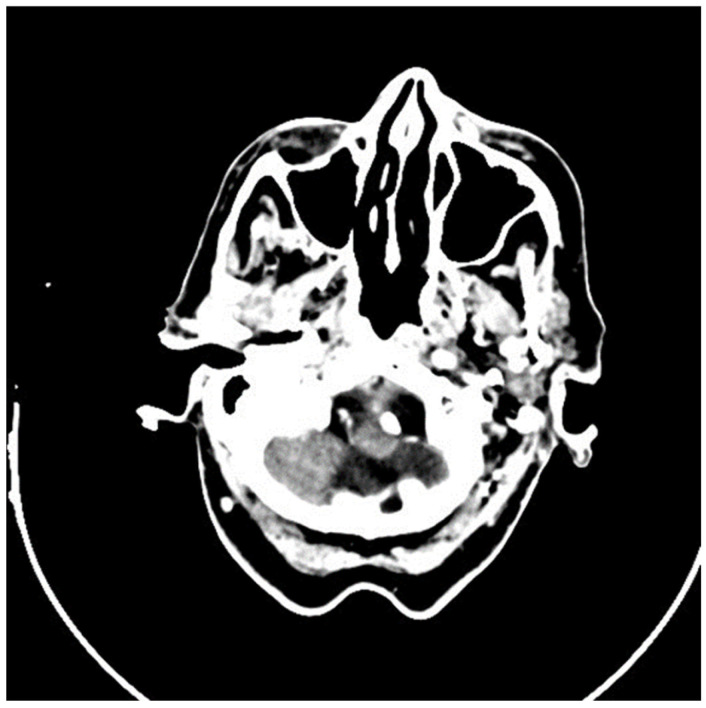
Preoperative CT imaging showing tumor involvement in the left nasal region.

To evaluate the extent of local invasion and to exclude regional or distant metastasis, a comprehensive imaging workup was performed. A head and neck computed tomography (CT) scan revealed soft tissue thickening of the left nasal wing, accompanied by nasal deviation, but without any evidence of osseous destruction (Figure 1). No cervical lymphadenopathy was detected. A neck ultrasound further confirmed the absence of suspicious lymph nodes. Additionally, an abdominal ultrasound and chest CT scan showed no signs of distant metastases.

The imaging findings supported the diagnosis of SEDC, demonstrating an infiltrative soft tissue mass without significant nodal involvement or distant spread. Notably, the absence of bony destruction later helped to distinguish SEDC from more aggressive variants of SCC, which often exhibit bone invasion at later stages.

### 2.3. Surgical Treatment

**Figure 2:**
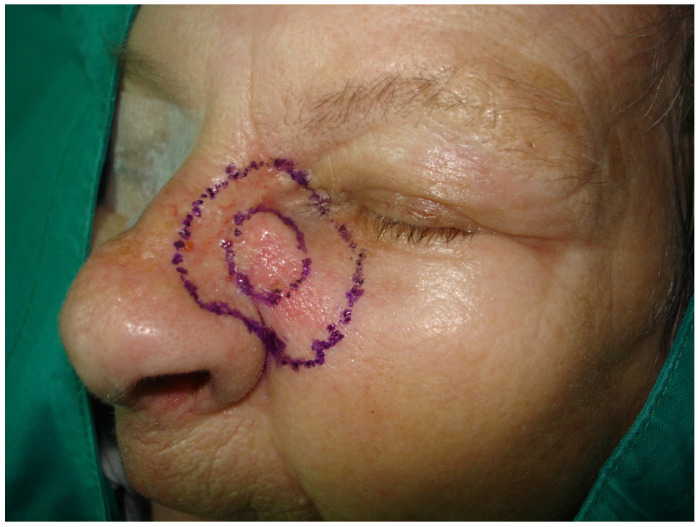
Preoperative plan for tumor resection.

**Figure 3:**
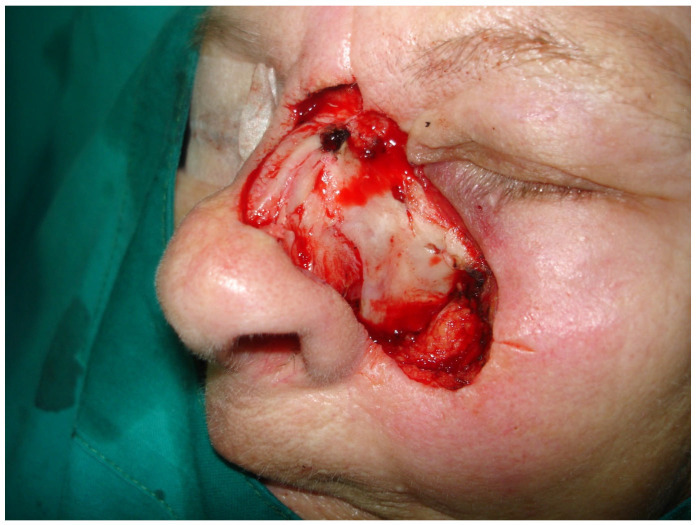
Radical resection of the soft tissues of the nose and left facial region.

Given the high-risk features observed in histology, a multidisciplinary tumor board discussion was conducted involving dermatopathologists, oncologists, and head and neck surgeons. The decision was made to proceed with radical surgical excision. Radical resection of the skin and soft tissues of the nose and the left side of the face was performed up to the nasal bones and maxillary bone, with intraoperative margin analysis (MMS) to ensure complete tumor removal (Figure 2 and Figure 3).

**Figure 4:**
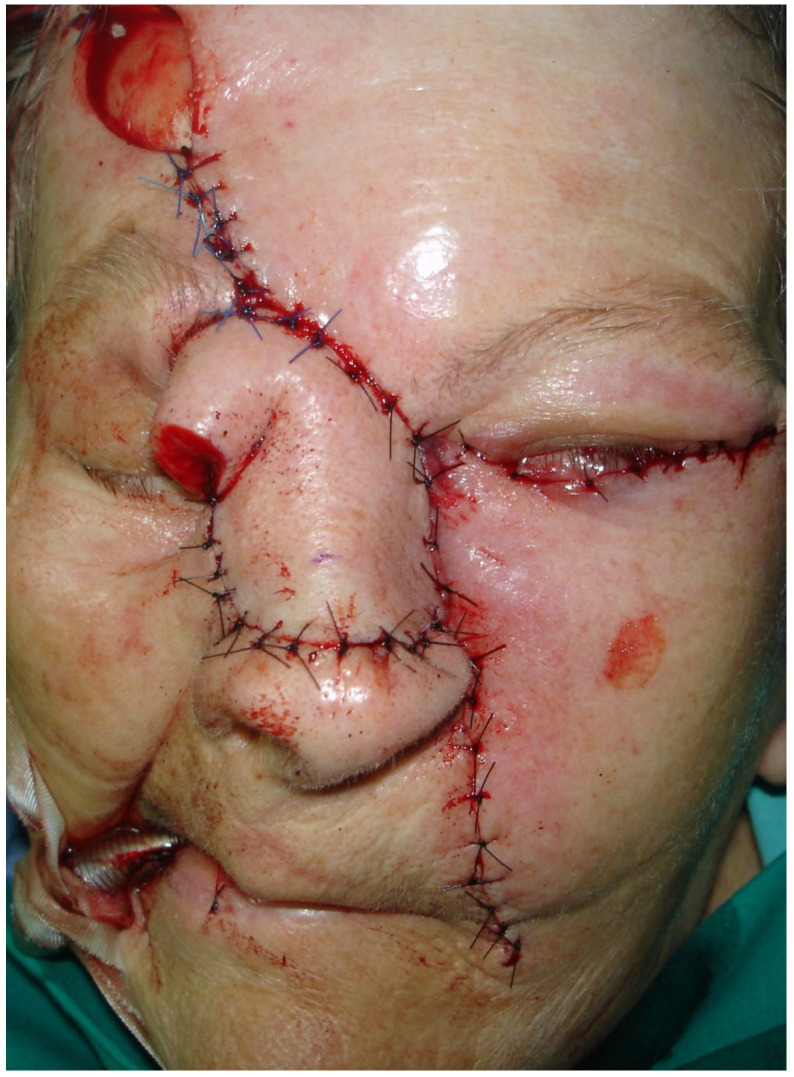
The residual defect of the middle and upper third of the nose and the midface region was reconstructed with a forehead flap and a cheek advancement flap on the left side of the face.

The residual defect of the middle and upper third of the nose and the midface region was reconstructed with a forehead flap and a cheek advancement flap on the left side of the face (Figure 4).

### 2.4. Histopathological Findings

**Figure 5:**
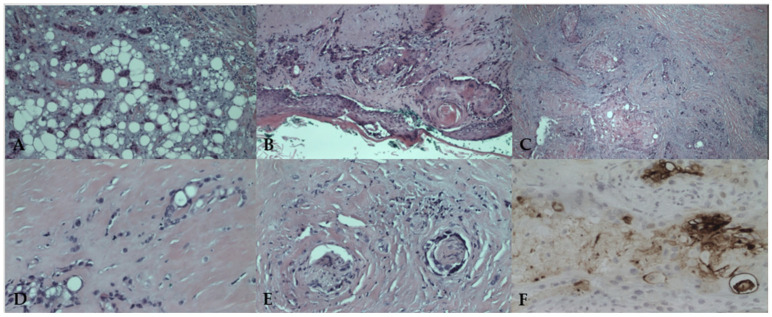
Histopathological analysis confirming squamoid eccrine ductal carcinoma: (**A**) poorly demarcated tumor infiltrating deep dermis and subcutaneous tissue (H&E, ×100); (**B**) tumor connection with the overlying epidermis and ulceration (H&E, ×100); (**C**) prominent squamoid differentiation and eccrine ducts (H&E, ×100); (**D**) tumor cells with intracytoplasmic vacuoles embedded in dense fibrous stroma (H&E, ×200); (**E**) perineural infiltration (H&E, ×200); (**F**) ductal differentiation confirmed with immunohistochemistry (CEA, ×200).

Histologic analysis confirmed the diagnosis of SEDC, measuring 15 mm in diameter, infiltrating the dermis, subcutaneous fatty tissue, cartilage, and transverse wound muscle, with both perivascular and perineural invasion (Figure 5A–F). The tumor demonstrated a biphasic pattern, with a squamoid component in superficial layers and infiltrative ductal differentiation in deeper layers. Immunohistochemical staining confirmed CEA positivity in ductal structures, aiding in differentiation from SCC. All surgical margins were free of tumor involvement.

### 2.5. Postoperative Course and Follow-Up

**Figure 6:**
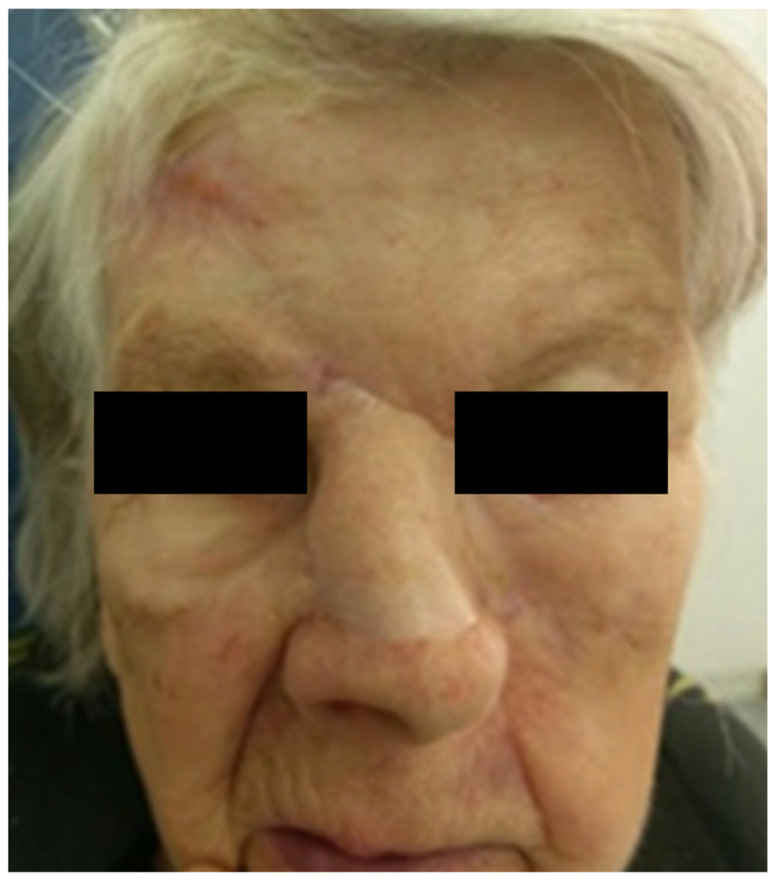
The patient 8 months after surgery.

The patient was referred for adjuvant radiotherapy, receiving a total dose of 62 Gy in 31 fractions to the tumor bed. She was regularly followed up with clinical examinations, CT scans, and ultrasounds (Figure 6).

**Figure 7:**
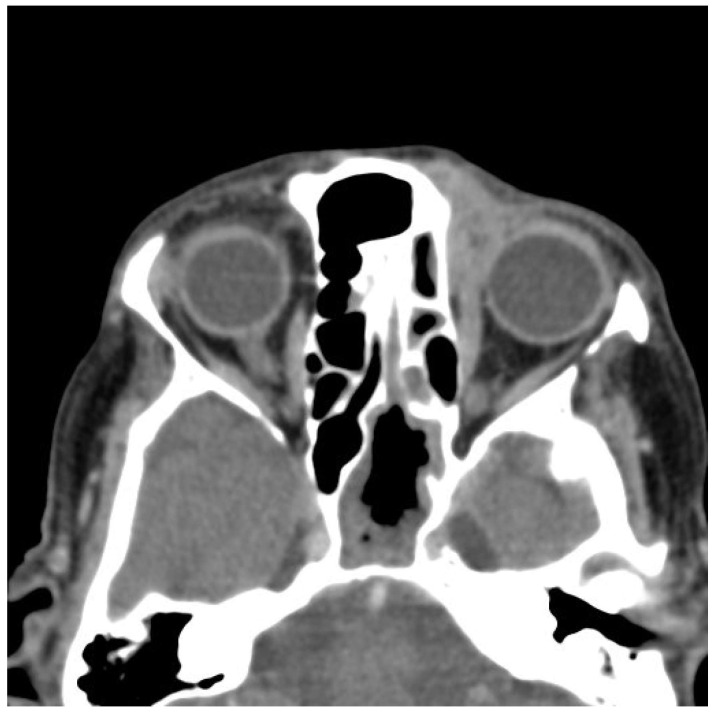
Local recurrence detected on follow-up CT scan.

Approximately 20 months after surgery, the patient noticed a small lesion in the region of the left medial canthus. A biopsy confirmed local recurrence of SEDC, and CT imaging demonstrated tumor extension into the left orbit, with infiltration of the medial rectus muscle and nasal bone (Figure 7).

Despite being offered exenteration of the orbit with nasal bone resection, the patient refused further surgical treatment. She received palliative care with analgesics. Then, 39 months after disease progression, she developed complications due to COVID-19, which ultimately led to her deterioration and passing. Although COVID-19 was not directly responsible for tumor progression, its impact on the patient’s overall health may have contributed to delayed intervention and worsening clinical status.

## 3. Discussion

### 3.1. Literature Review

As aforamentioned, SEDC is an exceptionally rare malignant adnexal tumor with significant clinical implications due to its potential for locoregional aggressiveness and metastatic spread [16]. The tumor predominantly arises in sun-exposed areas, particularly the head and neck, and is more commonly observed in elderly male patients, typically over 80 years old [2,4]. A recent systematic review by Saifi et al. [17] identified 92 cases of SEDC, with a mean patient age of 69 years. Reported tumor sizes range from 0.15 cm to 1.8 cm, with an average diameter of 0.43 cm [16]. Clinically, SEDC often presents as nodules or ulcerated plaques, which may be mistaken for SCC or other cutaneous neoplasms, leading to diagnostic delays [10,11]. Moreover, due to its nonspecific clinical presentation, SEDC is frequently subjected to incomplete biopsies (e.g., shave biopsies), which can also contribute to delayed diagnosis and adversely affect prognosis [13]. Table 1 and Table 2 present all previously described case reports and case series of SEDC in the head and neck region, along with their characteristics.

A narrative synthesis of the cases presented in Table 1 and Table 2 highlights that recurrence rates of SEDC remain alarmingly high, with a substantial proportion of cases experiencing local recurrence despite complete excision and adjuvant therapy [2,7,13]. Although less frequent, metastatic spread has also been documented in a notable number of reported cases. The most common sites of recurrence include the regional lymph nodes and, in more aggressive cases, distant metastases to the lungs and bones [4,8,21,34]. Additionally, PNI is a significant predictor of recurrence, as seen in multiple cases where the tumor infiltrated deep tissue structures despite histologically clear margins. The literature suggests that tumors with PNI are more likely to require multimodal treatment approaches, including both surgical excision and adjuvant radiotherapy, to optimize outcomes [2,7,11,22,28,33]. However, even with aggressive management, late recurrences have been observed, necessitating long-term surveillance [7,13,28,33]. Metastatic cases often follow an unpredictable course, with some patients exhibiting rapid progression despite early intervention. The presence of lymphovascular invasion (LVI) has been associated with a higher likelihood of systemic dissemination, particularly in cases where regional lymph nodes were positive at the time of diagnosis [7,20,28]. Saifi et al. [17] highlighted that SEDC is characterized by a recurrence rate of 17% and poor post-surgical 5-year survival rates of less than 30%. Their findings indicate that MMS may offer lower recurrence rates compared to wide local excision, though treatment approaches remain largely based on case reports and small case series. Adjuvant therapies, including radiotherapy and hormonal therapy, have been employed in metastatic cases, but their effectiveness remains uncertain [17]. Given these findings, clinicians should maintain a high index of suspicion for recurrence and metastasis, emphasizing close follow-up and imaging assessments post-treatment of SEDC.

### 3.2. Histopathological and Immunohistochemical Features

SEDC exhibits an infiltrative and malignant growth pattern with poorly demarcated margins, frequently extending beyond the surgical excision site [1,2]. The tumor displays a biphasic histopathological pattern, with squamous differentiation in superficial areas and ductal structures resembling eccrine differentiation in the deeper portions [35]. Due to its biphasic nature, superficial biopsies often lead to misdiagnosis as SCC since only the squamous component may be visible [23]. The immunohistochemical profile of SEDC is critical for differentiating it from SCC and other adnexal tumors. Positive staining for EMA and CEA in ductal areas, while being negative in squamoid regions, strongly suggests an eccrine origin [4,16]. Additional markers such as CK5/6 and p63 are useful in ruling out metastatic carcinoma and other malignancies with overlapping histological features [36]. In our case, initial biopsy findings were inconclusive, showing features suggestive of poorly differentiated squamous carcinoma or adnexal carcinoma with squamoid differentiation. Postoperative histological examination revealed a poorly demarcated, asymmetrical tumor infiltrating the deep dermis and subcutaneous tissue. The tumor was associated with an overlying ulcerated epidermis, while its superficial portion exhibited prominent squamoid differentiation, mimicking SCC. In the deeper layers, the tumor displayed a more infiltrative pattern, forming small nests and cords within dense fibrous stroma. Tumor cells demonstrated moderate atypia, eosinophilic cytoplasm, and large hyperchromatic nuclei, with some cells exhibiting intracytoplasmic vacuoles. The presence of ductal differentiation was confirmed immunohistochemically with CEA, aiding in differentiating SEDC from SCC. Additionally, perineural invasion was prominent, consistent with the tumor’s aggressive clinical behavior (Figure 5A–F).

While EMA and CEA remain the primary markers for eccrine differentiation, their specificity for SEDC has been questioned, as some cases of SCC with glandular differentiation may exhibit focal positivity. CK5/6 and p63, which are frequently expressed in SCC, can sometimes overlap with SEDC, necessitating a broader immunohistochemical panel. Emerging markers such as podoplanin (D2-40) and androgen receptor (AR) have been explored in distinguishing eccrine carcinoma subtypes, though their utility in SEDC remains unclear [37,38,39]. Recent literature has also explored the role of next-generation sequencing (NGS) and molecular profiling in distinguishing SEDC from other cutaneous adnexal carcinomas. While no specific genetic signature has been identified for SEDC, studies suggest that TP53, PIK3CA, and HRAS mutations may be implicated in adnexal malignancies, similar to SCC [37,40]. Incorporating molecular testing into routine diagnostics could enhance the accuracy of distinguishing SEDC from histopathologic mimickers and guide the development of targeted therapies. However, more research is required to establish standardized molecular markers for SEDC.

### 3.3. Differential Diagnosis

**Figure 8:**
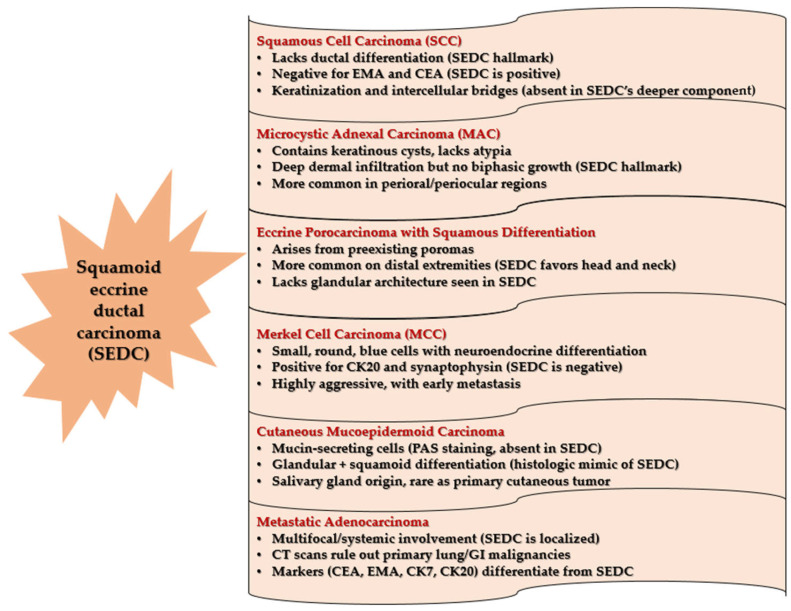
Differential diagnosis of squamoid eccrine ductal carcinoma (SEDC) based on histopathologic and immunohistochemical features (an original scheme reproduced from the available literature [2,4,7,15,16,36,41,42,43,44]).

The differential diagnosis for SEDC includes SCC, metastatic adenocarcinoma, microcystic adnexal carcinoma, porocarcinoma, mucoepidermoid carcinoma, and Merkel cell carcinoma (MCC) (Figure 8) [2,4,7,15,16,36,41,42,43,44].

SCC lacks the ductal differentiation seen in SEDC and typically expresses negative staining for EMA and CEA, which are useful distinguishing markers, while SEDC exhibits an infiltrative component with angulated basaloid and tubular structures within a desmoplastic stroma [2,4,40]. Microcystic adnexal carcinoma, another aggressive cutaneous adnexal tumor, differs from SEDC by exhibiting keratinous cyst formation, bland cytology, and an absence of prominent squamous components [43]. Porocarcinoma, although similar in morphology, generally arises in preexisting poromas and is more commonly located on the distal extremities [7]. In contrast, cutaneous mucoepidermoid carcinoma, which primarily originates from the salivary glands, features mucin-secreting cells detectable using periodic acid-Schiff (PAS) staining, aiding in differentiation from SEDC [41]. In our case, the presence of ductal differentiation, confirmed with CEA positivity, favored a diagnosis of SEDC over SCC. Additionally, metastatic adenocarcinoma was ruled out through chest and abdominal CT scans and ultrasound, confirming no evidence of primary malignancies elsewhere [42]. Given these challenges, adequate biopsy sampling that captures the deeper infiltrative component is crucial for accurate diagnosis. In our case, a definitive diagnosis was established only after complete excision and detailed histopathologic evaluation. Nowadays, molecular analysis has provided additional insight into the distinction between these tumors. MCC is strongly associated with Merkel cell polyomavirus (MCPyV) and demonstrates CK20 positivity in a perinuclear dot pattern, which is not observed in SEDC [43,45]. Furthermore, microcystic adnexal carcinoma (MAC) has been found to frequently harbor mutations in PTEN and NOTCH1, which are absent in SEDC, providing an avenue for molecular differentiation [46,47].

### 3.4. Diagnostics, Treatment, and Management

Due to the rarity of SEDC, there are no standardized treatment guidelines, and management is largely extrapolated from other cutaneous adnexal carcinomas. Imaging techniques such as magnetic resonance imaging (MRI) and CT scans have been utilized in select cases to assess the extent of primary, recurrent, or metastatic SEDC [26,48]. Given the tumor’s potential for both local recurrence and distant metastasis, routine follow-up is highly recommended. In our patient, MMS was performed with intraoperative margin control, followed by adjuvant radiotherapy (62 Gy in 31 fractions) due to high-risk features, including perineural invasion and deep tissue involvement. Surgical excision remains the primary treatment modality, with MMS being the preferred approach for facial lesions, as it allows for precise margin control while preserving healthy tissue [8,14]. In cases with regional lymph node involvement, lymphadenectomy is recommended. However, given the tumor’s high recurrence rate, long-term surveillance is crucial. A review of treatment outcomes found that among 8 patients treated with MMS and 16 patients who underwent conventional excision, recurrence rates were 12.5% and 31.25%, respectively [7]. The recurrence timeline of 20 months postoperatively observed in our case aligns with prior literature, which reports a median recurrence period of 14 to 24 months [4,7]. While adjuvant radiotherapy has been employed in cases where the tumor demonstrated neurovascular invasion, failed multiple excisions, or recurred after surgery, its role in delaying recurrence remains uncertain, as some cases have shown disease progression despite treatment [24]. For instance, some studies suggest that PNI in certain aggressive cancers similar to SEDC (such as SCC) may confer intrinsic radioresistance, necessitating close follow-up and potential multimodal strategies [49,50].

Chemotherapy has not been routinely explored in SEDC treatment due to its rarity and limited data. However, systemic chemotherapy regimens used in other aggressive adnexal carcinomas, such as cisplatin-based therapy or taxane combinations, may be considered in metastatic cases. However, conventional chemotherapy has shown limited benefit in metastatic eccrine carcinoma [51,52]. Emerging treatments, like immunotherapy, have demonstrated promising results in similar malignancies [53]. Targeted therapies, including EGFR inhibitors or immune checkpoint inhibitors, could offer potential treatment avenues, though their effectiveness in SEDC has not been studied. Given the role of HRAS and PIK3CA mutations in some adnexal malignancies, PI3K pathway inhibitors or MEK inhibitors may hold future promise [54,55].

Despite achieving clear surgical margins, in this case, local recurrence occurred after 20 months, presenting as a small lesion in the medial canthal region. The patient’s refusal of orbital exenteration underscores the ethical principle of patient autonomy, which allows individuals to make informed decisions about their healthcare, even when these decisions may not align with medical recommendations. Balancing this autonomy with the healthcare provider’s duty of beneficence—acting in the patient’s best interest—can be challenging, particularly in cases involving aggressive malignancies where the standard treatment is extensive surgery [56]. Orbital exenteration is a radical procedure associated with significant cosmetic and functional consequences, often leading to substantial psychosocial impacts. Patients may experience an altered body image and diminished quality of life post-surgery. Therefore, when a patient declines such an intervention, it is imperative to explore alternative, less invasive treatments that align with their preferences and maintain their quality of life [57]. In scenarios where patients refuse extensive surgical procedures, palliative approaches become essential. These may include localized radiotherapy to manage symptoms and control tumor progression, thereby preserving the patient’s comfort and dignity. Additionally, emerging treatments, such as immunotherapy, have shown promise in managing certain aggressive cancers (such as SCC) without resorting to disfiguring surgeries, offering potential avenues to address the disease while respecting the patient’s wishes [58]. Ultimately, respecting patient autonomy requires a patient-centered approach that carefully considers the individual’s values, preferences, and quality-of-life considerations. Engaging in open, empathetic discussions about the risks and benefits of all available treatment options ensures that patients are well-informed and supported in their decision-making process.

Our patient ultimately succumbed to COVID-19 complications, raising questions about the interplay between viral infections and aggressive cutaneous malignancies. While no direct correlation between COVID-19 and SEDC progression has been documented, immunosuppressive states and inflammatory responses induced by viral infections could contribute to overall disease burden and worsen outcomes. Further research is required to elucidate the impact of systemic infections on adnexal tumor behavior [59,60]. Given the earlier mentioned significant recurrence and metastasis risks of SEDC, future research should explore the potential benefits of systemic therapies, such as targeted molecular treatments or immunotherapy, particularly in high-risk patients. In cases where patients decline extensive surgical interventions, such as in this case, alternative palliative approaches should be considered to balance oncologic control with patient quality of life.

## 4. Conclusions

SEDC is a rare, aggressive, and potentially underdiagnosed malignancy of the sweat glands, characterized by infiltrative growth, deep tissue invasion, and a high incidence of perineural infiltration. Despite complete surgical excision, local recurrence remains a frequent challenge, highlighting the need for vigilant post-treatment monitoring. Although traditionally classified as a low-grade malignancy, its capacity for recurrence and metastatic spread suggests that SEDC may be more appropriately categorized as an intermediate-grade tumor. Given its histologic overlap with SCC, accurate diagnosis requires a thorough histopathologic and immunohistochemical evaluation. The absence of universally accepted treatment guidelines and the limited understanding of its pathophysiology underscore the need for further research to establish evidence-based management strategies. Multidisciplinary collaboration and heightened clinical surveillance are particularly crucial for immunosuppressed patients, who may face an increased risk of poor prognosis. Raising awareness among clinicians and pathologists is essential to ensuring early recognition and intervention, ultimately improving patient outcomes. By advancing diagnostic accuracy and optimizing therapeutic approaches, the medical community can enhance the prognosis of this rare and challenging neoplasm. Long-term follow-up remains imperative to monitor for recurrence and metastatic progression, reinforcing the need for ongoing clinical and scientific efforts to better understand and manage SEDC.

## Figures and Tables

**Table 1:** Reported case reports of squamoid eccrine ductal carcinoma in the head and neck region and their characteristics in the available literature up to 1 January 2025.

Author, Year [Reference Number]	Gender/Age	Past Medical History	Site of the Tumor	Size (cm)	Presence of PNI and LVI	Management, Follow-Up, and Outcome
Wong et al., 1996 [2]	M/81	NA	Ear	≤2.5	PNI	Excision with 3 recurrences within 36 mo
Kim et al., 2005 [18]	F/30	Not remarkable	Neck	2.5	NA	Negative CT, Mohs, no recurrence at 5 mo
Terushkin et al., 2010 [19]	M/63	Chronic lymphocytic leukemia	Cheek	2.7	No	Mohs, no recurrence at 10 mo
Perkins et al., 2011 [20]	M/72	Multiple SCCs and esophageal adenocarcinoma	Ear	NA	LVI	Mohs, follow-up NA
Jung et al., 2012 [8]	M/53	Not remarkable	Scalp	2.6	NA	CT, excision, fine needle aspiration with metastasis to lymph node at 5 mo, excision and lymph node dissection
Kim et al., 2012 [21]	M/53	NA	Scalp	2.6	NA	CT, excision, fine needle aspiration with metastasis to lymph node at 5 mo, excision, and lymph node dissection
Ranasinghe et al., 2013 [22]	M/65	NA	Nose	0.7	PNI	Mohs, follow-up NA
Chan et al., 2016 [23]	M/85	Multiple nonmelanoma skin cancers and superficial spreading melanoma, myasthenia gravis	Scalp	NA	No	Excision, follow-up NA
Saraiva et al., 2016 [13]	F/72	Not remarkable	Nose	NA	NA	Excision 2× and RT for recurrence at 5 mo, no recurrence at 23 mo
Graham et al., 2017 [24]	M/80	NA	Forehead	NA	NA	Mohs, no recurrence at 24 mo
Sharma et al., 2018 [25]	M/50	NA	Scalp	1	NA	No recurrence at 5 mo
Rovesti et al., 2018 [11]	M/75	Colon cancer	Temple	2	PNI	Mohs, no recurrence at 12 mo
Lobo-Jardim et al., 2018 [10]	F/76	Bowen disease	Nose	NA	NA	Excision, follow-up NA
Yim et al., 2019 [26]	M/80	Not remarkable	Ear	1.5	NA	CT scan, excision, no recurrence at 9 mo
Bayramoğlu and Ünal, 2020 [5]	M/79	Not remarkable	Scalp	1.2	No	Local excision, follow-up NA
Phan et al., 2020 [27]	M/92	Thrombocytopenia, infiltrative BCC of the nose	Temple	2 × 2.2	Few foci of PNI	Local excision and watch-and-wait approach, no recurrence at 6 mo
Rownose et al., 2021 [28]	F/41	Not remarkable	Scalp	4	PNI and LVI	Wedge excision biopsy, suggested RT and ChT refused; died 3 mo after
Patel et al., 2022 [29]	M/76	SCC of the right naris, right nasal ala and scalp	Eyelid	2.5 × 1.5	No	Mohs stage 2, no recurrence at 27 mo
Kweon et al., 2024 [30]	M/84	Not remarkable	Ear auricle	NA	LVI	Parotidectomy, selective neck dissection, no recurrence at 12 mo
Chan et al., 2024 [31]	F/88	NA	Temple	2	NA	Mohs, follow-up NA
Eghtedari, Lin and Kim, 2024 [32]	F/82	Not remarkable	Cheek	NA	No	Mohs, no recurrence at 12 mo
Špiljak et al. [this case]	F/80	Not remarkable	Left nasal region	1 × 1.5	PNI	Mohs, adjuvant RT; recurrence after 20 mo; declined further surgery; died of COVID-19 39 mo after disease progression

Abbreviations: M = male; F = female; PNI = perineural invasion; LVI = lymphovascular invasion; NA = not available; mo = months; SCC = squamous cell carcinoma; RT = radiotherapy; CT = computed tomography; BCC = basal cell carcinoma; ChT = chemotherapy. Some cases in Table 1 lack specific details regarding tumor size, follow-up outcomes, or treatment response due to incomplete data in the original case reports. This limitation reflects the rarity of SEDC and variability in documentation across studies.

**Table 2:** Reported case series of squamoid eccrine ductal carcinoma in the head and neck region and their characteristics in the available literature up to 1 January 2025.

Author, Year [Reference Number]	Number of Cases	Gender/Mean Age	Past Medical History	Site of the Tumor	Mean Size (cm)	Presence of PNI and LVI	Management, Follow-Up, and Outcome
Frouin et al., 2015 [7]	7	6 F and 1 M/81.3	BCC (2×), SCC (2×), Bowen disease (2×), SSM (1×), breast cancer—radiation surgery (1×), kidney transplantat recipient (1×), liver transplantat recipient (1×)	3× cheek, 2× nose, 1× forehead, 1× canthus	3.45 (3× NA)	4× PNI and 4× LVI	Excision (3×), 2× excision (2×), 5× excision with enucleation, amputation and RT (1×), Mohs (1×), no recurrence at 44–156 mo (5×); recurred at 32 mo (1×), died from SEDC at 42 mo (1×)
Van der Horst, 2016 [4]	23	7 F and 16 M/68.4	NA	Cheek (5×), forehead (4×), ear (4×), scalp (3×), nose (3×), neck (2×), temple (1×), lip (1×)	1.045	NA	No recurrence at 6–51 mo (12×), recurrence at 7–20 mo and no recurrence at 13–50 mo (2×), recurrence at 24 mo (1×), lymph node metastasis and no recurrence at 99 mo—(1×), lymph node metastasis at 8 mo and recurrence at 14 mo—died at 32 mo (2×), died at 7–32 mo (3×), follow-up NA (2×)
Svoboda et al., 2021 [33]	5	1 F and 4 M/80.8	Active hepatitis B infection (1×), Crohn disease treated with azathioprine and history of melanoma (1×), chronic lymphocitic leukemia (1×), HIV and polymiositis treated with tacrolimus and intravenous immunoglobulin (1×)	Scalp (3×), eyebrow (1×), forehead (1×),	2.5 × 2.2	PNI (4×)	Excision (5×), adjuvant RT (2×), no recurrence at 6.5–18 mo (4×), newly identified case (1×)
Honorato et al., 2024 [34]	5	5 M/68	Kidney transplant recipients (3×)	Eyebrow (1×), temporal region (1×), forehead (1×), cervical region (1×), scalp (1×)	NA	PNI (3×) and LVI (1×)	Surgical excision (4×), NA (1×), no recurrence at 21–35 mo (2×), recurrence at 36 mo (1×); recurrence, lymph node and lung metastasis at 22 mo resulting in death (1×)

Abbreviations: F = female; M = male; PNI = perineural invasion; LVI = lymphovascular invasion; NA = not available; mo = months; BCC = basal cell carcinoma; SCC = squamous cell carcinoma; SSM = superficial spreading melanoma; SEDC = squamoid eccrine ductal carcinoma; RT = radiotherapy; HIV = human immunodeficiency virus. Some cases in Table 2 lack specific details regarding tumor size, follow-up outcomes, or treatment response due to incomplete data in the original case reports. This limitation reflects the rarity of SEDC and variability in documentation across studies.

## Data Availability

The authors confirm that the data supporting the findings of this study are available within the article.

## Abbreviations

The following abbreviations are used in this manuscript:

SEDC	Squamoid eccrine ductal carcinoma.
SCC	Squamous cell carcinoma.
EMA	Epithelial membrane origin.
CEA	Carcinogenic antigen.
MMS	Mohs micrographic surgery.
PNI	Perineural invasion.
CT	Computed tomography.
LVI	Lymphovascular invasion.
D2-40	Podoplanin.
AR	Androgen receptor.
NGS	Next-generation sequencing.
MCC	Merkel cell carcinoma.
PAS	Periodic acid-Schiff.
MCPyV	Merkel cell polyomavirus.
MAC	Mycrocystic adnexal carcinoma.
MRI	Magnetic resonance imaging.

## References

[1] Wick M.R., Swanson P.E. (1991). Cutaneous Adnexal Tumors. A Guide to Pathological Diagnosis.

[2] Wong T.Y., Suster S., Mihm M.C. (1997). Squamoid Eccrine Ductal Carcinoma. Histopathology.

[3] Brenn T. (2015). Malignant Sweat Gland Tumors: An Update. Adv. Anat. Pathol..

[4] van der Horst M.P., Garcia-Herrera A., Markiewicz D., Martin B., Calonje E., Brenn T. (2016). Squamoid Eccrine Ductal Carcinoma: A Clinicopathologic Study of 30 Cases. Am. J. Surg. Pathol..

[5] Bayramoğlu Z., Ünal B. (2020). Squamoid Eccrine Ductal Carcinoma: A Rare Case Report. Eur. Res. J..

[6] Bellisario J.A., Lunardhi A., Huynh K., Iguh C., Stohl H. (2024). Squamoid Eccrine Ductal Carcinoma: An Unusual Diagnosis in a Pregnant Patient. Cureus.

[7] Frouin E., Vignon-Pennamen M.D., Balme B., Cavelier-Balloy B., Zimmermann U., Ortonne N., Carlotti A., Pinquier L., André J., Cribier B. (2015). Anatomoclinical Study of 30 Cases of Sclerosing Sweat Duct Carcinomas (Microcystic Adnexal Carcinoma, Syringomatous Carcinoma and Squamoid Eccrine Ductal Carcinoma). J. Eur. Acad. Dermatol. Venereol..

[8] Jung Y.H., Jo H.J., Kang M.S. (2012). Squamoid Eccrine Ductal Carcinoma of the Scalp. Korean J. Pathol..

[9] Liang C.A., Busam K.J., Nehal K.S. (2011). Microcystic Adnexal Carcinoma Associated with Multiple Benign Syringomatous Proliferations: A Report of Two Cases. Dermatol. Surg..

[10] Lobo-Jardim M.M., Souza B.C., Kakizaki P., Valente N.Y.S. (2018). Dermoscopy of Squamoid Eccrine Ductal Carcinoma: An Aid for Early Diagnosis. An. Bras. Dermatol..

[11] Rovesti M., Satolli F., Ricci R., Manuguerra R., Zucchi A., Gandolfi M., Gianfaldoni S., Claudio F., Wollina U., Tchernev G. (2018). Once in a Blue Moon … Rare Adnexal Tumor: From the Clinical and Videodermoscopic Aspects to the Mohs Surgery and the Histological Diagnosis. Open Access Maced. J. Med. Sci..

[12] Lim M.M., Macdonald J.A. (2022). Squamoid Eccrine Ductal Carcinoma: Treatment and Outcomes. Am. J. Dermatopathol..

[13] Saraiva M.I., Vieira M.A., Portocarrero L.K., Fraga R.C., Kakizaki P., Valente N.Y. (2016). Squamoid Eccrine Ductal Carcinoma. An. Bras. Dermatol..

[14] Clark S., Young A., Piatigorsky E., Ravitskiy L. (2013). Mohs Micrographic Surgery in the Setting of Squamoid Eccrine Ductal Carcinoma: Addressing a Diagnostic and Therapeutic Challenge. J. Clin. Aesthet. Dermatol..

[15] van der Horst M.P.J., Brenn T. (2017). Update on Malignant Sweat Gland Tumors. Surg. Pathol. Clin..

[16] Elder D.E., Massi D., Scolyer R.A., Willemze R. (2018). WHO Classification of Skin Tumors.

[17] Saifi B., Maalouf M., Hasan A., Fadel D., Kassab M., Emmanuel N. (2025). Squamoid Eccrine Ductal Carcinoma: A Systematic Review. Curr. Dermatol. Rep..

[18] Kim Y.J., Kim A.R., Yu D.S. (2005). Mohs Micrographic Surgery for Squamoid Eccrine Ductal Carcinoma. Dermatol. Surg..

[19] Terushkin E., Leffell D.J., Futoryan T., Cowper S., Lazova R. (2010). Squamoid Eccrine Ductal Carcinoma: A Case Report and Review of the Literature. Am. J. Dermatopathol..

[20] Perkins A.C., Goldberg D., Maloney M.E. (2011). Primary Eccrine Ductal Carcinoma Masquerading as Metastatic Squamous Cell Carcinoma. J. Am. Acad. Dermatol..

[21] Kim J.W., Jeon M.K., Kang S.J., Sun H. (2012). Surgical Management of Recurrent Squamoid Eccrine Ductal Carcinoma of the Scalp. J. Craniofacial Surg..

[22] Ranasinghe A., Rytina E., Flanagan N., Jenkins R., Ha T. (2013). Squamoid Eccrine Ductal Carcinoma: A Rare Adnexal Tumor. J. Am. Acad. Dermatol..

[23] Chan H., Howard V., Moir D., Dyall-Smith D. (2016). Squamoid Eccrine Ductal Carcinoma of the Scalp. Australas. J. Dermatol..

[24] Graham P., Krach K. (2017). Squamoid Eccrine Ductal Carcinoma: A Unique Diagnostic and Therapeutic Challenge. J. Am. Acad. Dermatol..

[25] Sharma B., Malhotra P., Bhardwaj M., Bhartiya R., Kumari N., Agrawal P., Rajnish K. (2018). Squamoid Eccrine Ductal Carcinoma: A Diagnostic Dilemma. Ann. Pathol. Lab. Med..

[26] Yim S., Lee Y.H., Chae S.W., Kim W.S. (2019). Squamoid Eccrine Ductal Carcinoma of the Ear Helix. Clin. Case Rep..

[27] Phan K., Kim L., Lim P., Cheung K. (2020). A Case Report of Temple Squamoid Eccrine Ductal Carcinoma: A Diagnostic Challenge Beneath the Tip of the Iceberg. Dermatol. Ther..

[28] Rownose C.S., Mohamad Saupi M.S., Sharif S.Z., Lah N.A.S.N. (2021). Aggressive Scalp and Sternal Lesion: A Presentation of Rare Case of Metastatic Eccrine Carcinoma. Ann. Med. Surg..

[29] Patel N., Alabiad C.R., Wick M.R., Elgart G.W., Tang V.D., Abou Khzam R.A., Dubovy S.R. (2022). Squamoid Eccrine Ductal Carcinoma of the Eyelid: Clinicopathologic Correlation of a Case. Ophthalmic Plast. Reconstr. Surg..

[30] Kweon H.T., Lee C.M., Yeo C.D., Lee E.J. (2024). A Case of Squamoid Eccrine Ductal Carcinoma of the Auricle Mimicking Perichondritis. Ear Nose Throat J..

[31] Chan C.X., Sriharan A., LeBlanc R.E., Guill M.A., Glass J.S., Momtahen S. (2024). Squamoid Eccrine Ductal Carcinoma: Clinical, Histological and Immunohistochemical Features. Dermatol. Online J..

[32] Eghtedari M., Lin M.J., Kim L. (2024). Mohs Surgery for Squamoid Eccrine Ductal Carcinoma. J. Cutan. Aesthetic Surg..

[33] Svoboda S.A., Rush P.S., Garofola C.J., Grider D.J., Prickett K.A., Phillips M.A. (2021). Squamoid Eccrine Ductal Carcinoma. Cutis.

[34] Honorato C.M.A., Carrascoza G.G., Abed N.M., Moya F.G. (2024). Squamoid Eccrine Ductal Carcinoma: Series of Five Cases of a Rare Tumor. An. Bras. Dermatol..

[35] Jacob J., Kugelman L. (2018). Squamoid Eccrine Ductal Carcinoma. Cutis.

[36] Ibrahim Y.L., Lambert S., Kazakov D.V., Kaya G. (2018). An Unusual Morphological Presentation of Cutaneous Squamous Cell Carcinoma Mimicking Microcystic Adnexal Carcinoma: A Diagnostic Pitfall. Dermatopathology.

[37] Macagno N., Sohier P., Kervarrec T., Pissaloux D., Jullie M.-L., Cribier B., Battistella M. (2022). Recent Advances on Immunohistochemistry and Molecular Biology for the Diagnosis of Adnexal Sweat Gland Tumors. Cancers.

[38] Compton L.A., Murphy G.F., Lian C.G. (2015). Diagnostic Immunohistochemistry in Cutaneous Neoplasia: An Update. Dermatopathology.

[39] Tuffaha M.S.A., Guski H., Kristiansen G. (2023). Markers and Immunoprofile of Skin Tumors. Immunohistochemistry in Tumor Diagnostics.

[40] Harms P.W., Hovelson D.H., Cani A.K., Omata K., Haller M.J., Wang M.L., Arps D., Patel R.M., Fullen D.R., Wang M. (2016). Porocarcinomas Harbor Recurrent HRAS-Activating Mutations and Tumor Suppressor Inactivating Mutations. Hum. Pathol..

[41] López V., Rubio M., Santonja N., Jordá E. (2010). Primary Cutaneous Mucoepidermoid Carcinoma. Am. J. Dermatopathol..

[42] Sauerborn D., Vidaković B., Baranović M., Longoria M.A., Alcalá J.I. (2012). Metastatic Adenocarcinoma in the Head and Neck Region. Adenocarcinoma: Pathogenesis, Treatment, and Prognosis.

[43] Cardoso J.C., Calonje E. (2015). Malignant Sweat Gland Tumors: An Update. Histopathology.

[44] McKissack S.S., Wohltmann W., Dalton S.R., Miletta N.R. (2018). Squamoid Eccrine Ductal Carcinoma: An Aggressive Mimicker of Squamous Cell Carcinoma. Am. J. Dermatopathol..

[45] Busam K.J., Jungbluth A.A., Rekthman N., Coit D., Pulitzer M., Bini J., Arora R., Hanson N.C., Tassello J.A., Frosina D. (2009). Merkel Cell Polyomavirus Expression in Merkel Cell Carcinomas and Its Absence in Combined Tumors and Pulmonary Neuroendocrine Carcinomas. Am. J. Surg. Pathol..

[46] Louveau B., Nakouri I., Jouenne F., Baroudjian B., Sadoux A., Da Meda L., Osio A., Reinhart F., Robert J., Herms F. (2024). Genomic Profiling of a Skin Adnexal Carcinomas Cohort Using a Comprehensive High-Throughput Sequencing Approach. Br. J. Dermatol..

[47] Harms P.W., Runge M., Chan M.P., Liu C.J., Qin Z., Worden F., Robinson D.R., Chinnaiyan A.M., Mclean S.A., Harms K.L. (2024). Squamoid Eccrine Ductal Carcinoma Displays Ultraviolet Mutations and Intermediate Gene Expression Relative to Squamous Cell Carcinoma, Microcystic Adnexal Carcinoma, and Porocarcinoma. Mod. Pathol..

[48] Wang B., Jarell A.D., Bingham J.L., Bonavia G.H. (2015). PET/CT Imaging of Squamoid Eccrine Ductal Carcinoma. Clin. Nucl. Med..

[49] Hutchinson M.K.N.D., Mierzwa M., D’Silva N.J. (2020). Radiation Resistance in Head and Neck Squamous Cell Carcinoma: Dire Need for an Appropriate Sensitizer. Oncogene.

[50] Stevenson M.L., Criscito M.C., Wilken R., Doudican N.A., Bain E.E., Parashar B., Carucci J.A. (2020). Use of Adjuvant Radiotherapy in the Treatment of High-Risk Cutaneous Squamous Cell Carcinoma with Perineural Invasion. JAMA Dermatol..

[51] De Iuliis F., Amoroso L., Taglieri L., Vendittozzi S., Blasi L., Salerno G., Lanza R., Scarpa S. (2014). Chemotherapy of Rare Skin Adnexal Tumors: A Review of Literature. Anticancer Res..

[52] Płachta I., Kleibert M., Czarnecka A.M., Spałek M., Szumera-Ciećkiewicz A., Rutkowski P. (2021). Current Diagnosis and Treatment Options for Cutaneous Adnexal Neoplasms with Apocrine and Eccrine Differentiation. Int. J. Mol. Sci..

[53] Fadhil M., Lochhead A., Trinh H., Brungs D. (2023). Metastatic Ductal Eccrine Adenocarcinoma with Excellent Response to Immunotherapy. Case Rep. Oncol..

[54] Wee S., Jagani Z., Xiang K.X., Loo A., Dorsch M., Yao Y.M., Sellers W.R., Lengauer C., Stegmeier F. (2009). PI3K Pathway Activation Mediates Resistance to MEK Inhibitors in KRAS Mutant Cancers. Cancer Res..

[55] Janku F., Wheler J.J., Westin S.N., Moulder S.L., Naing A., Tsimberidou A.M., Fu S., Falchook G.S., Hong D.S., Garrido-Laguna I. (2012). PI3K/AKT/mTOR Inhibitors in Patients with Breast and Gynecologic Malignancies Harboring PIK3CA Mutations. J. Clin. Oncol..

[56] Pirotte B.D., Benson S. Refusal of Care. https://www.ncbi.nlm.nih.gov/books/NBK560886/.

[57] Sokoya M., Cohn J.E., Kohlert S., Lee T., Kadakia S., Ducic Y. (2019). Considerations in Orbital Exenteration. Semin. Plast. Surg..

[58] McLean L.S., Lim A.M., Webb A., Cavanagh K., Thai A., Magarey M., Fox C., Kleid S., Rischin D. (2022). Immunotherapy to Avoid Orbital Exenteration in Patients with Cutaneous Squamous Cell Carcinoma. Front. Oncol..

[59] Borg T.M., Eisold J., Miyanjo Y., Pappa E. (2023). The Effect of COVID-19 on Surgical Management of Skin Cancers of the Head, Face and Neck in Elderly Patients. Skin Health Dis..

[60] Sengar M., Chinnaswamy G., Ranganathan P., Ashok A., Bhosale S., Biswas S., Chaturvedi P., Dhamne C., Divatia J., D’Sa K. (2022). TMH COVID-19 Action Group. Outcomes of COVID-19 and Risk Factors in Patients with Cancer. Nat. Cancer.

